# Bilateral Pleural Effusion in Cryptogenic Organizing Pneumonia: A Case Report and Review of Atypical Presentations

**DOI:** 10.7759/cureus.84390

**Published:** 2025-05-19

**Authors:** Hussain A Alwesaibi, Abdullah H Albin Saad, Mohammed S Almulaify, Majid G Alqatari

**Affiliations:** 1 Department of Internal Medicine, Dammam Medical Complex, Dammam, SAU; 2 Department of Pulmonary Medicine, Dammam Medical Complex, Dammam, SAU

**Keywords:** community-acquired pneumonia (cap), cryptogenic organizing pneumonia (cop), ground-glass opacity, idiopathic cryptogenic organizing pneumonia, multifocal pulmonary consolidation, non-resolving pleural effusion, non-resolving pneumonia, oral corticosteroids, pleural effusion, secondary organizing pneumonia

## Abstract

Cryptogenic organizing pneumonia (COP) is a rare form of idiopathic interstitial lung disease that often presents with non-specific respiratory symptoms and radiographic findings that mimic infectious pneumonia. Due to these overlapping clinical features, COP is frequently misdiagnosed, leading to delays in appropriate management.

We report the case of a 23-year-old female patient who presented with dyspnea, productive cough, and fever. Despite receiving broad-spectrum antimicrobial therapy, there was no clinical or radiographic improvement. Imaging revealed bilateral pulmonary opacities and pleural effusions, and diagnostic bronchoscopy with transbronchial biopsy confirmed organizing pneumonia. Secondary causes, including infections, autoimmune diseases, and malignancy, were excluded. The patient started on oral prednisolone, resulting in significant improvement in both symptoms and radiological findings.

This case underscores the importance of considering COP in patients with non-resolving pneumonia, particularly when there is no response to adequate antibiotic therapy. A combination of radiological assessment, histopathological confirmation, and exclusion of secondary causes is essential for accurate diagnosis. Clinicians should maintain a high index of suspicion for COP in such scenarios, as timely diagnosis and initiation of corticosteroid therapy are critical for symptom resolution and prevention of disease progression.

## Introduction

Cryptogenic organizing pneumonia (COP) is a form of idiopathic, diffuse interstitial lung disease that was previously referred to as bronchiolitis obliterans organizing pneumonia (BOOP) [1,2]. It is characterized by an inflammatory response and the presence of granulation tissue plugs within the lumens of small airways, alveoli, and alveolar ducts [3]. Although relatively common in the inpatient setting, COP is frequently misdiagnosed as infectious pneumonia due to overlapping clinical features such as cough, dyspnea, and fever [3].

COP lacks pathognomonic radiographic findings. However, common imaging features include bilateral, peripheral, and diffuse alveolar or interstitial opacities [3]. These findings may mimic a range of other pulmonary conditions, complicating the diagnostic process and underscoring the importance of histopathological confirmation [3].

We report the case of a young female patient who presented with bilateral pleural effusions and was initially treated for community-acquired pneumonia. The condition was later classified as non-resolving pneumonia, and a definitive diagnosis of COP was established following biopsy.

## Case presentation

We report a case of a 23-year-old female patient from Saudi Arabia with no significant past medical history who presented to the emergency department with complaints of dyspnea, productive cough, and generalized fatigue that had been present for the past three days. The patient also reported a documented fever at home, reaching 40°C, along with reduced oral intake over the preceding days. She denied experiencing nausea, vomiting, headache, dizziness, skin rash, or other systemic symptoms.

There was no history of similar previous episodes, no history of recent travel, no contact with sick individuals, no recent viral illness, no recent antibiotic use, and no family history of rheumatologic disease. The patient also denied previous history of joint pain, dry eyes, or dry mouth.

On physical examination, the patient was conscious, alert, and oriented to time, place, and person. She was not in respiratory distress and had a respiratory rate of 20 breaths per minute. Her general appearance appeared ill, with dry oral mucosa. Vital signs were as follows: blood pressure 104/59 mmHg, heart rate 102 beats per minute, temperature 37.7°C, and oxygen saturation of 99% on room air. Chest auscultation revealed bilateral coarse crackles, more prominent in the right lower lung zone. Abdominal examination was unremarkable, with a soft, non-tender abdomen. Cardiovascular examination revealed normal heart sounds with no evidence of lower limb edema or signs suggestive of deep vein thrombosis.

Initial laboratory investigations revealed mild anemia, thrombocytopenia, mild hypokalemia, and elevated C-reactive protein, with normal liver enzyme levels (see Table 1).

**Table 1:** Initial laboratory findings on admission. Values in bold represent values that are above or below the normal levels.

Test	Result	Normal range	Units
WBC (white blood cell)	4.4	4.0-11.0	×10⁹ per litre (×10⁹/L)
Hemoglobin	10.7	13.5-17.5	grams per decilitre (g/dl)
Red blood cells (RBC)	4.21	4.3-6.5	10^12^/L
Hematocrit (HCT)	32.3	37-47	%
Mean corpuscular volume (MCV)	76.7	80-94	Femtoliters (fL)
Mean corpuscular hemoglobin (MCH)	25.2	27-32	Picograms (pg)
Mean corpuscular hemoglobin concentration (MCHC)	32.8	32-36	g/dl
Red cell distribution width - coefficient of variation (RDW-CV)	14.6	11.5-14.5	%
Platelet	115	140-450	10^9^/L
Serum creatinine	53	62-115	Micromoles per liter (µmol/L)
Sodium (Na⁺)	139	135–145	Millimoles per litre (mmol/L)
Potassium (K⁺)	3.4	135–145	mmol/L
PH	7.44	7.35-7.45	-
Carbone monoxide	37	35-45	mmHg
Bicarbonate	26	22-28	mmol/L
AST(aspartate aminotransferase)	35	10-40	International units per litre (IU/L)
ALT (alanine aminotransferase)	23	7-56	IU/L
Total bilirubin	9	3-21	µmol/L
Conjugated bilirubin	4	0-7	µmol/L
GGT (gamma-glutamyl transferase)	60	8-61	IU/L
ALP (alkaline phosphate)	67	40-129	IU/L
Erythrocyte sedimentation rate (ESR)	28	0-20	mm/h
C-reactive protein (CRP)	17.05	0-3.3	milligrams per liter (mg/L)
Reverse transcription polymerase chain reaction (RT-PCR) testing for Coronavirus disease-19 (COVID-19)	Negative	Negative	-
Reverse transcription polymerase chain reaction (RT-PCR) testing for Influenza A,B	Negative	Negative	-
Human immune deficiency virus (antibody/antigen)	Undetected	Undetected	-

**Figure 1:**
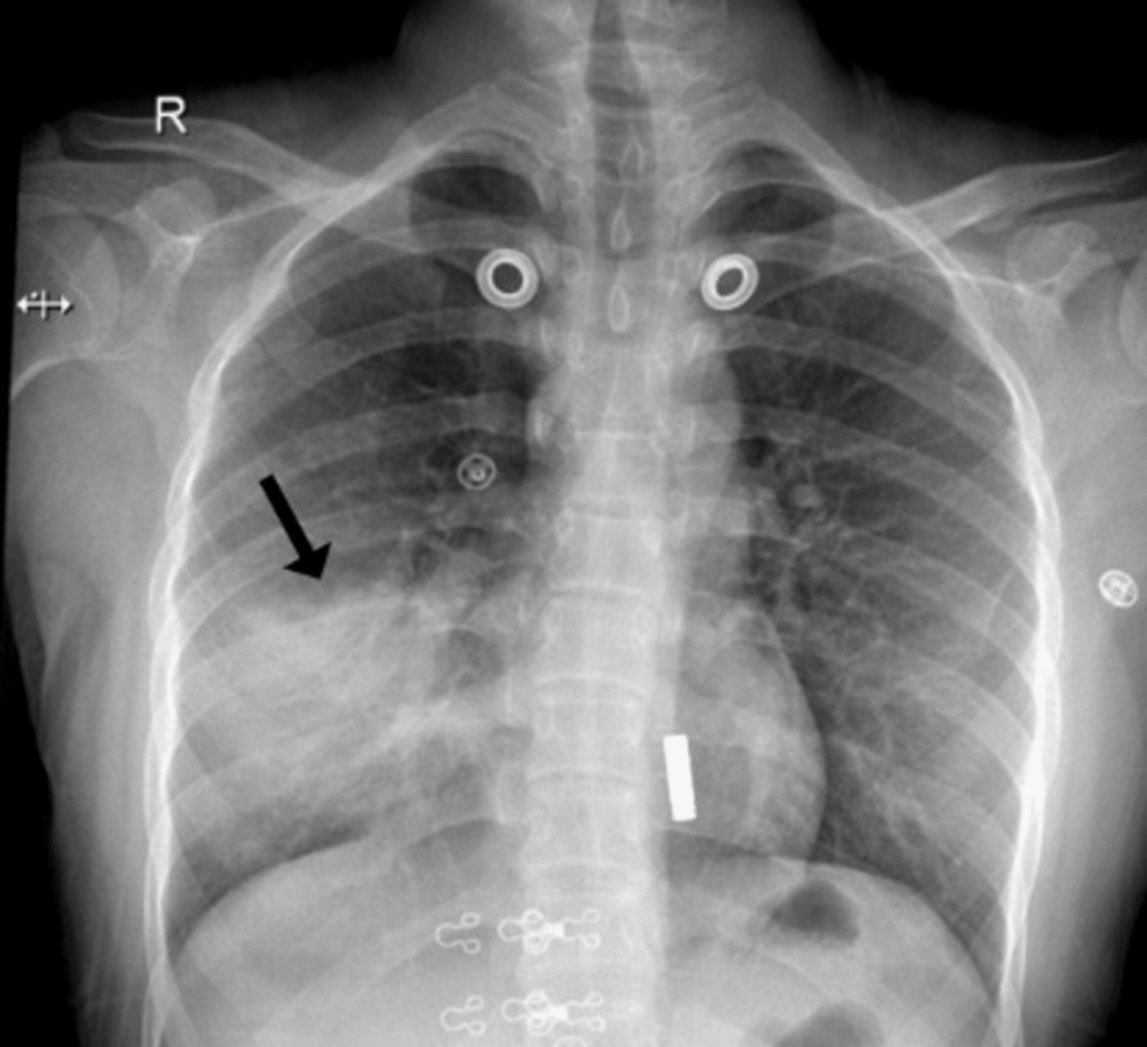
Initial chest radiograph showing right lower zone heterogeneous opacity representing air space disease consistent with lobar pneumonia (black arrow).

The patient was admitted to the general medical ward with a provisional diagnosis of community-acquired pneumonia (lobar pneumonia), based on her clinical presentation - which included dyspnea, productive cough, and fever - and initial chest radiographic findings. A chest radiograph revealed a heterogeneous opacity in the right lower lung zone (Figure 1).

Empirical antibiotic therapy was initiated with intravenous ceftriaxone 2 g once daily and oral azithromycin 500 mg once daily. Potassium supplementation was also administered to correct the mild hypokalemia.

**Figure 2:**
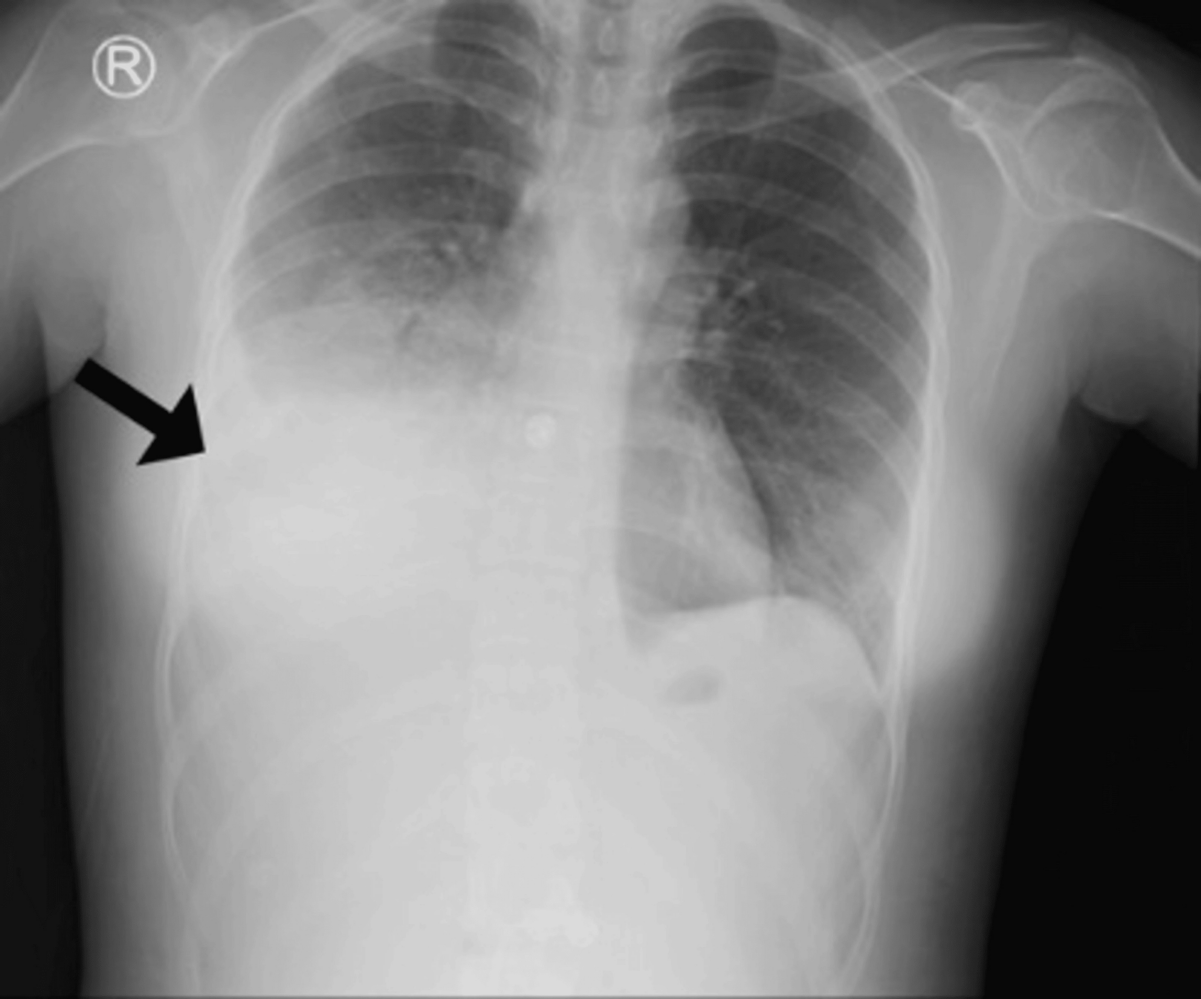
Follow-up chest radiograph showed obliteration of the right costophrenic angle most likely plural effusion with new opacities involving the right lower and middle lung zones (Black arrow).

Despite two days of treatment, the patient continued to experience fever and a productive cough, with no clinical improvement. Furthermore, she began requiring supplemental oxygen at 2 liters per minute via nasal cannula. A follow-up chest radiograph was obtained, revealing obliteration of the right costophrenic angle and new opacities involving the right lower and middle lung zones (Figure 2).

Ceftriaxone was discontinued, and intravenous piperacillin/tazobactam (4.5 g every six hours) was initiated.

**Figure 3:**
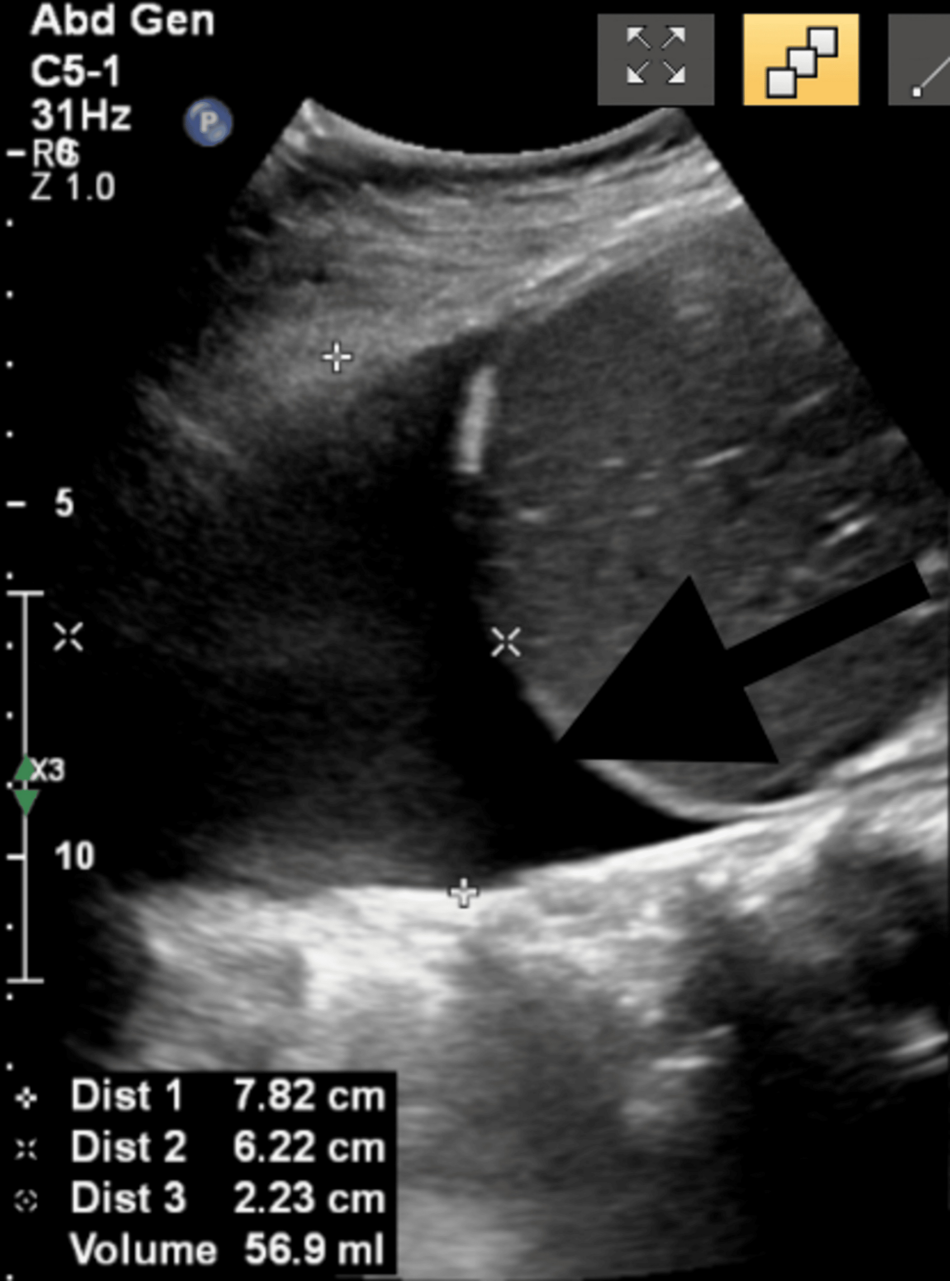
A chest ultrasonography revealing a right-sided anechoic pleural effusion without septations or solid components (black arrow).

A chest ultrasonography was performed, which revealed a right-sided anechoic pleural effusion without septations or solid components (Figure 3).

Under full aseptic technique, a pigtail catheter was inserted on the right side, resulting in the initial drainage of approximately 400 mL of turbid fluid. The pleural fluid was sent for analysis, which revealed an exudative effusion based on Light’s criteria (Table 2).

**Table 2:** Results of pleural fluid analysis.

Test	Result	Normal range	Units
Culture and sensitivity	No growth	No growth	-
Glucose	68	60-100	mg/dl
Albumin	16	1-3	g/L
Total protein	26	1-3	g/L
Lactate dehydrogenase (LDH)	1464	<200	units per litre (U/L)
pH	8	7.6-7.64	-
White blood cells (WBCs)	296	<1000	cells per microliter (cells/μL)
Red blood cells (RBCs)	2000	0	cells/µL

**Figure 4:**
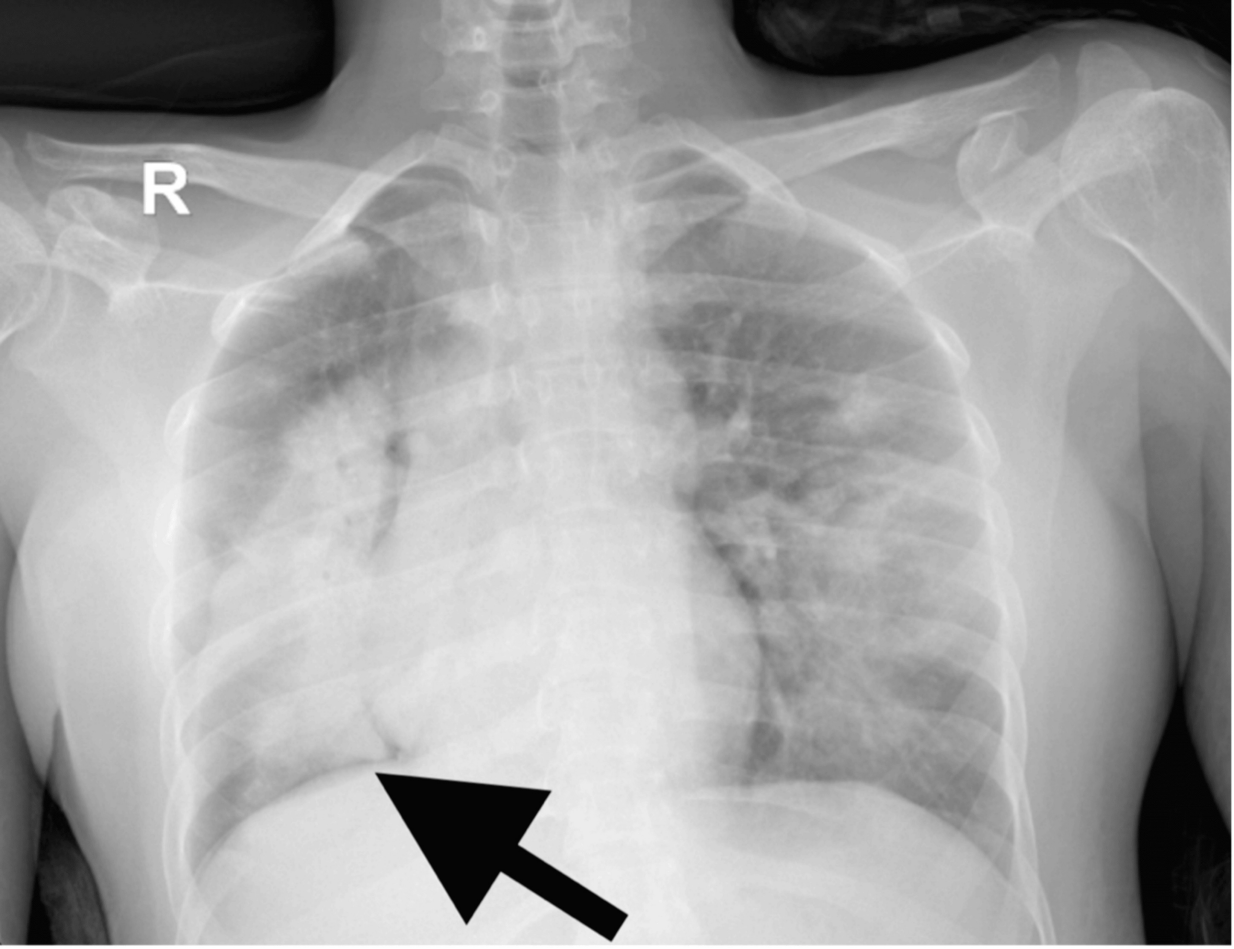
A Follow-up chest radiograph after removing the pigtail demonstrated full lung expansion with resolution of plural effusion in the right side with a residual right-sided opacity (black arrow).

Following pleural fluid drainage, linezolid was added to the antibiotic regimen to cover a possible superimposed *Staphylococcus aureus* infection. The pigtail catheter was removed after five days, and a follow-up chest radiograph demonstrated full lung expansion with a residual right-sided opacity (Figure 4).

**Figure 5:**
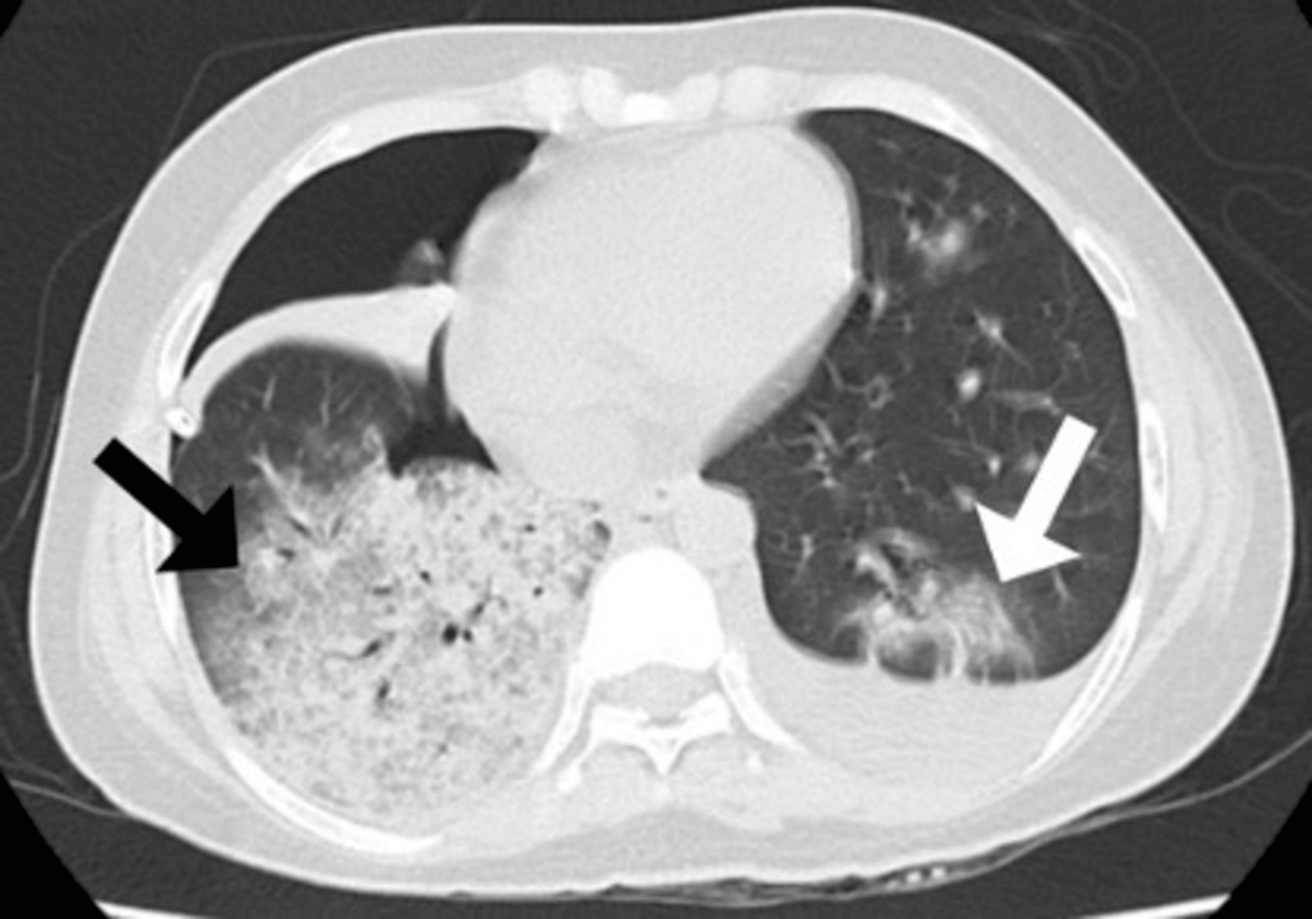
A follow-up chest CT scan revealing bilateral moderate airspace disease characterized by areas of consolidation and ground-glass opacities, more pronounced on the right side. Additionally, the scan showed a mild left-sided pleural effusion (white arrow) and a mild right-sided pneumothorax and mild plural effusion (black arrow).

Four days later, a follow-up chest computed tomography (CT) scan was performed, revealing bilateral moderate airspace disease characterized by areas of consolidation and ground-glass opacities, more pronounced on the right side. The scan also showed a mild left-sided pleural effusion, a mild right-sided pneumothorax, and small right-sided pleural effusion (Figure 5).

The patient continued to experience febrile episodes, with documented temperatures reaching 38°C, along with persistent respiratory symptoms. Two sets of blood cultures and a sputum culture were obtained, all of which showed no microbial growth. Notably, a new left-sided pulmonary opacity developed, which was not present on the initial chest radiograph. Additionally, there was evidence of re-accumulation of pleural effusion.

Given the lack of response to broad-spectrum antibiotics and the progression of radiographic findings, the patient underwent bronchoscopy. Bronchoalveolar lavage (BAL) samples were sent for bacterial, fungal, tuberculosis (acid-fast bacilli (AFB)), cytological analysis, and cell count. A transbronchial lung biopsy was also obtained for histopathological examination. Cytological analysis of the BAL sample was negative for malignancy, and all cultures, including AFB, were negative. An autoimmune workup was also performed, with negative results (Table 3).

**Table 3:** Autoimmune serology results.

Test	Result	Normal range	Units
Antinuclear antibody (ANA)	Negative	No growth	-
Anti dsDNA Antibody	Negative	60-100	-
C3 complement	0.82	0.74-1.42	g/L
C4 complement	0.24	0.24-0.46	g/L
Anti smith	Negative	-	-
Anti–cyclic citrullinated peptide (anti-CCP)	Negative	-	-
Rheumatoid factor	Negative	-	-

Given the strong clinical suspicion of organizing pneumonia and imaging findings - supported by the lack of clinical and radiographic improvement despite a full course of antimicrobial therapy, along with the presence of recurrent pleural effusion - a decision was made to initiate corticosteroid therapy. After discussing the differential diagnosis, treatment rationale, and potential side effects of corticosteroids with the patient, oral prednisolone was started.

The patient maintained adequate oxygen saturation on room air and was discharged on oral prednisolone 40 mg once daily. At the time of discharge, she continued to report a persistent cough.

**Figure 6:**
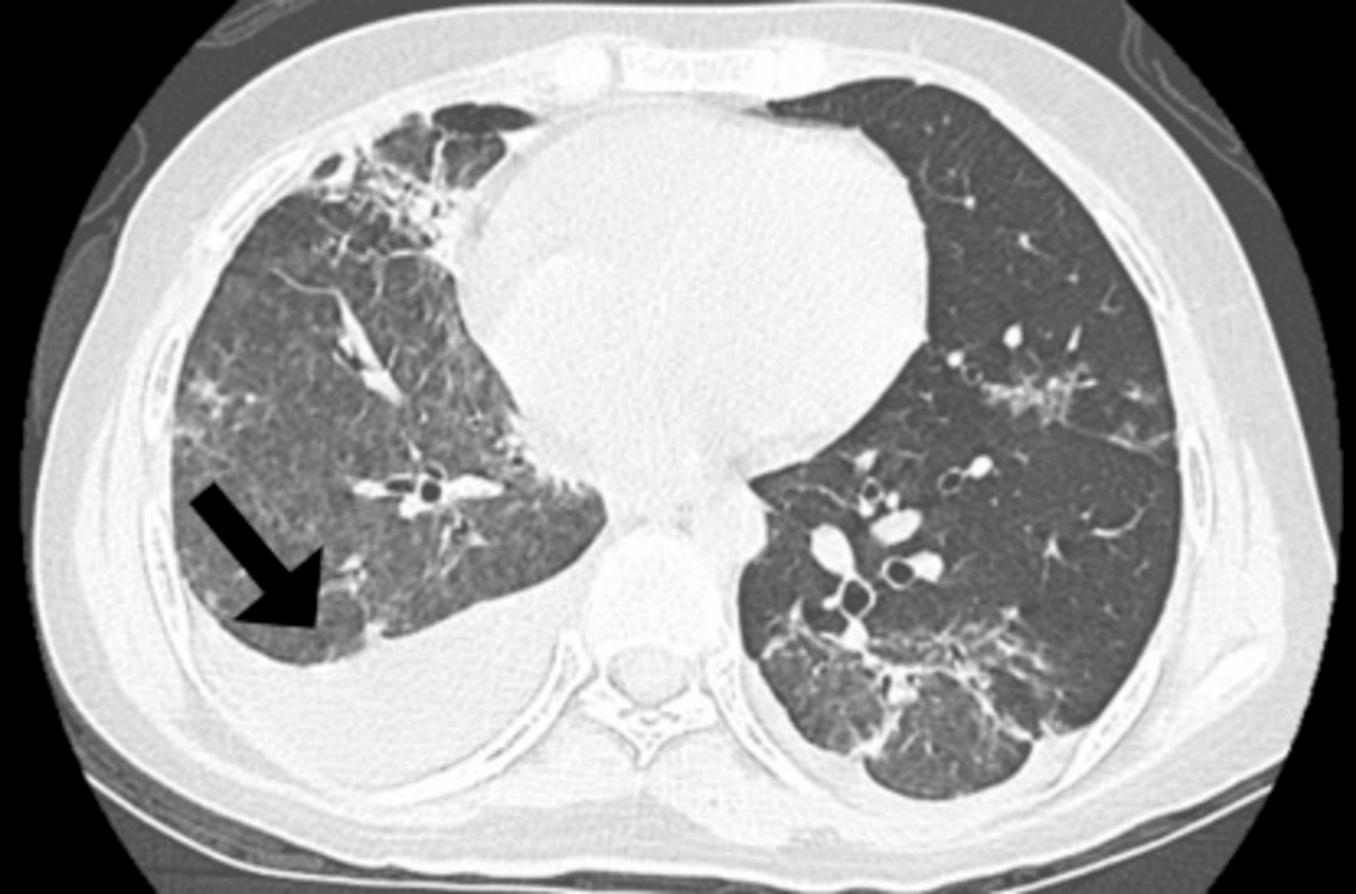
HRCT during follow-up showed improvement in the bilateral airspace disease, with residual moderate bilateral ground-glass opacities, consolidation in the middle lobe, and a right-sided pleural effusion (black arrow). HRCT: high-resolution computed tomography

During outpatient follow-up, histopathological analysis of the transbronchial biopsy revealed lung tissue with fibrosis and chronic inflammation consistent with organizing pneumonia. A high-resolution computed tomography (HRCT) scan performed after two weeks during follow-up showed improvement in the bilateral airspace disease, with residual moderate bilateral ground-glass opacities, consolidation in the middle lobe, and a right-sided pleural effusion (Figure 6).

**Figure 7:**
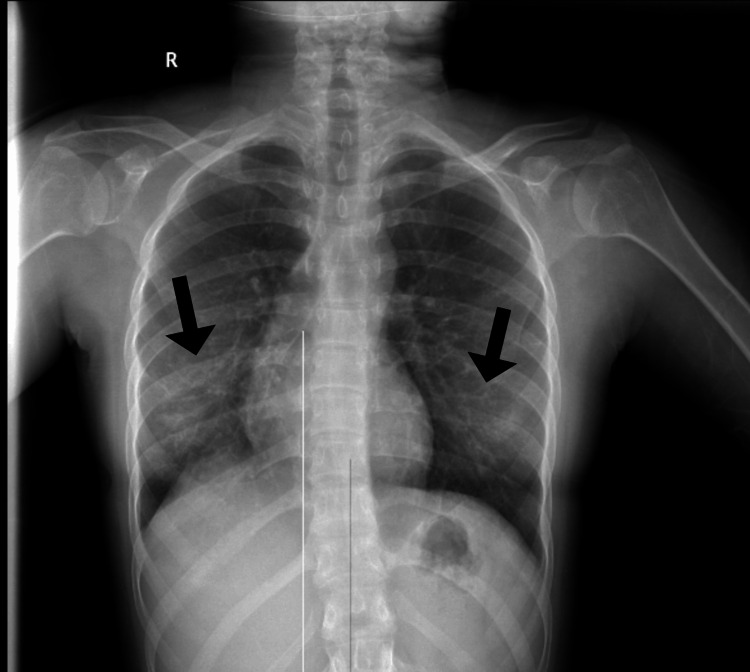
A chest radiograph obtained six weeks post-discharge, following HRCT, demonstrated complete resolution of the bilateral opacities and resolution of the right-sided pleural effusion (black arrows). HRCT: high-resolution computed tomography

The patient continued on oral prednisolone. Her symptoms improved markedly following the initiation of corticosteroid therapy, with significant resolution of dyspnea and cough, as reported during outpatient follow-up. A chest radiograph obtained six weeks post-discharge, following the HRCT, demonstrated complete resolution of the bilateral opacities and resolution of the right-sided pleural effusion (Figure 7).

## Discussion

COP typically presents with non-specific respiratory symptoms such as cough and fever. When there is a history of prolonged antibiotic therapy without significant clinical improvement - as in our patient, who received multiple courses of antimicrobials with no meaningful response - COP should be considered in the differential diagnosis [4].

Organizing pneumonia (OP), referred to as COP when no identifiable cause is found, is a subtype of interstitial lung disease. It represents a non-specific response to lung injury and is characterized histologically by granulation tissue plugs within the lumens of small airways, alveolar ducts, and alveoli. The inflammatory response is mediated by fibroblasts and myofibroblasts embedded in a connective tissue matrix [4].

The diagnosis of COP requires the exclusion of secondary causes, including infections, medications, surgery, radiotherapy, autoimmune diseases, and malignancies [4]. In our patient, secondary causes were thoroughly ruled out. Autoimmune serologies were negative, and there were no clinical signs or symptoms suggestive of connective tissue disease. The patient denied any recent medication or antibiotic use prior to hospitalization, and malignancy was excluded based on negative cytology from BAL samples.

Infectious causes, including COVID-19 and influenza, have been associated with organizing pneumonia in previous literature [5-7]. However, both were excluded in our case using RT-PCR testing for COVID-19 and reverse transcriptase polymerase chain reaction (RT-PCR) testing for influenza A and B.

Radiographically, bilateral asymmetrical consolidations are the most frequently observed finding in OP, reported in approximately 80-95% of cases, typically with a subpleural distribution [8,9]. This pattern was evident in our patient. Ground-glass opacities, the second most common radiographic feature, were also observed in this case [8,9]. Bilateral pleural effusions, though relatively uncommon in OP, are more frequently associated with secondary organizing pneumonia than with COP. They have been reported in approximately 10-35% of OP cases [8,9]. Our patient developed bilateral pleural effusions, which initially responded to drainage but reaccumulated before resolving following corticosteroid therapy.

Corticosteroids are the mainstay of treatment for COP. In a review of 12 studies involving 160 patients, treatment with prednisone resulted in complete remission in 60%, partial improvement in 27%, no response in 14%, and mortality in 6% of cases [10]. Clinical improvement is often observed within a few days of initiating corticosteroids, with significant radiological and symptomatic responses typically occurring within a few weeks [11,12]. However, relapses are not uncommon. A systematic review reported a relapse rate of 36%, often occurring during tapering or after discontinuation of therapy. Common side effects of corticosteroids include weight gain, myopathy, and osteoporosis [13].

Our patient was started on oral prednisolone 40 mg daily, resulting in marked clinical and radiographic improvement. During follow-up every two weeks, the dose was gradually tapered 5 mg weekly to reach 10 mg daily without evidence of relapse. The patient did report weight gain in three weeks duration during corticosteroid course, a known side effect of corticosteroid therapy [13].

## Conclusions

Cryptogenic organizing pneumonia is a rare but important differential diagnosis in patients presenting with persistent respiratory symptoms and non-resolving pneumonia despite adequate antimicrobial therapy. This case highlights the diagnostic challenge COP poses, especially when initial radiographic findings mimic infectious etiologies. A high index of suspicion, exclusion of secondary causes, and histopathological confirmation are essential for accurate diagnosis. Prompt initiation of corticosteroid therapy can lead to significant clinical and radiological improvement, as demonstrated in our patient. Early recognition and appropriate treatment of COP are crucial to prevent unnecessary interventions and optimize patient outcomes.
